# Family health history and pharmacogenomics show cross generation premature amitriptyline discontinuation is associated with CYP2C19 loss of-function enrichment

**DOI:** 10.1038/s43856-025-01156-3

**Published:** 2025-11-13

**Authors:** Emma F. Magavern, Gabriel Marengo, Maia Megase, Damian Smedley, Mark J. Caulfield

**Affiliations:** William Harvey Research Institute, Queen Mary University of London, London, UK

**Keywords:** Pharmacogenetics, Adverse effects

## Abstract

**Background:**

Genetics influence medication response yet integrating family health history (FHH) of medication response with pharmacogenomics remains underexplored. The objective of this study was to examine cross-generational medication exposure patterns and assess the utility of FHH for medication response using electronic health records.

**Methods:**

Genes & Health (G&H) data from Bangladeshi and Pakistani participants were analysed for parent-offspring trios with medication exposure. Premature discontinuation of amitriptyline was defined as discontinuation within three months. Logistic regression and Fisher’s exact test explored the relationship between parental discontinuation, offspring discontinuation, and *CYP2C19* loss-of-function variants.

**Results:**

13% of offspring prescriptions overlap with parental prescriptions, primarily short-term treatments (antibiotics, vaccines, steroids). In 96 trios, cross-generational amitriptyline exposure is observed. Parental discontinuation does not predict offspring discontinuation (*p* = 0.275, OR 1.70). However, offspring with two-generation histories of early discontinuation are significantly enriched for *CYP2C19* predicted poor metabolizers (38% vs. 10.5%, *p* = 0.049, OR 4.86).

**Conclusions:**

FHH of medication response can highlight individuals enriched for pharmacogenomic variants. This is a clinically useful finding, which may flag psychiatric patients with a two-generation history of early amitriptyline discontinuation or intolerance as a priority for pharmacogenomic testing. This study demonstrates the potential of integrating FHH into clinical pharmacogenomics for actionable insights.

## Plain language summary

It is routine medical practice to ask about family history of medical problems. Though we know that some risk of reacting badly to a medication is also inherited it is not common practice to look for clues in family history when deciding which medicine might be best suited for a patient. This study looked at how commonly participants were prescribed medication that a parent had been prescribed and found that 13% of medication prescribed to a child had also been prescribed to a parent. The commonly used antidepressant amitriptyline was prescribed to a child and a parent in 96 families within this study. The authors examined patterns of amitriptyline prescriptions to see if medication use was predictive of inherited characteristics known to impact on medication response. They found that a parent stopping the medication quickly did not predict that the child would also do so. However, in families where both the parent and the child stopped taking amitriptyline within 3 months, the children were nearly 5 times more likely to be slow amitriptyline metabolisers (predicted from DNA to break down the medicine slowly). This shows that using routine prescribing data from families can highlight offspring with high genetic risk of an atypical response to medication.

## Abstract (web summary)

Magavern, Marengo et al. examine cross-generational medication exposure patterns and assess the utility of family health history for medication response using electronic health records. They find that offspring with two-generation histories of early amitriptyline discontinuation were significantly enriched for CYP2C19 predicted poor metabolizers.

## Introduction

The use of Family Health History (FHH) in clinical practice and creation of pedigrees of those family members who are and are not affected by the disease of interest are well understood in rare diseases.^1^ FHH is also routinely integrated in common disease clinical assessments, such as a clinical history upon a presentation with a suspected acute coronary syndrome.^2^ Interindividual variability in response to medication is known to be problematic, with many individuals either not getting the intended benefit of a medication or suffering from an adverse drug event (ADR).^3^ There is a genetic component to this variability in response, and the relationship between genetic variants and drug response is known as pharmacogenomics (PGx).^4^ However, there are also many non-genetic contributions to medication response and empiric studies teasing out the genetic from non-genetic factors are complicated by many factors, such as uncertain exposure (medication adherence is known to be around 50%), dynamic physiologic changes that may not be accounted for (i.e. acute liver or kidney disfunction), environmental, dietary and supplement use factors, and limited phenotypic characterisation of medication response in electronic health record codes. Additionally, the large number of study participants needed to detect even moderate effect changes due to genetics in the context of the above limitations is a significant barrier.^5^

Although FHH is commonly used in rare and common disease diagnostic pathways and is clearly of potential utility in predicting drug response where there is a genetic component with a strong effect size, it has not been integrated into routine clinical use. There are some examples in which it would seem to be clinically valuable to do so, for example in the case of aminoglycoside induced hearing loss, known to be associated with mitochondrial variants in *MT-RNR1.*^6^ It would be reasonable to ask, before prescribing an aminoglycoside antibiotic, if any relatives on the maternal side have lost their hearing after having an antibiotic, but this is not reflected in practice and has not been suggested as far as we are aware.

There is the potential to explore use of family history in low-resource settings where genetic testing isn’t available for PGx. It could potentially also help to target limited resources for PGx to highest impact cases.

Though FHH has ended up being the purview of disease diagnosis, the origins of elucidation of genetic contribution to rare severe ADRs is also in FHH and pedigree analysis. A prominent example is the case of malignant hyperthermia, where the genetic cause was identified due to a severe ADR in response to anaesthetic agents across multiple generations of a family.^7^ However, there has been little written and a paucity of discussion around integration of medication response phenotype with FHH.^8^

Unlike disease risk stratification, the use of FHH data to assist in predicting response to medication is dependent on medication exposure. Therefore, a natural question would be what is the empiric evidence around medication exposure overlap across generations in a modern context? Current research has not asked or answered this question, that we are aware of. Electronic health care records can offer opportunities to extract and organise family data on response to medication, particularly if novel AI tools were to be utilised. However, it has not been demonstrated if such an approach may have utility to predict medication response or highlight individuals who should undergo PGx testing.

This study seeks to fill a gap in evidence by illustrating cross-generational patterns of medication exposure and provide proof-of-concept for utility of FHH data on medication response which can be opportunistically taken from electronic health records. We describe, as far as we are aware, cross-generational prescription medication exposure for the first time in a British South Asian cohort of 600 parent–offspring trios with linked genetic and primary-care prescription data. We find that parental early discontinuation of amitriptyline does not predict offspring discontinuation (*p* = 0.275, OR 1.70). However, when both parent and child independently discontinue within three months, offspring are significantly enriched for CYP2C19 poor-metabolizer genotypes (38% vs. 10.5%, OR 4.86, *p* = 0.049). These findings demonstrate that a two-generation history of early medication discontinuation can serve as a pragmatic marker to flag individuals for targeted pharmacogenomic testing and more personalised prescribing strategies.

## Methods

Genes & Health (G&H) is a longitudinal community genetics study focusing on participants of self-declared Bangladeshi and Pakistani ancestry aged 18 and older.^9^ DNA samples collected during recruitment have undergone genotyping array analysis (GSA) and a subgroup have had whole exome sequencing (WES). Participants provided consent for linkage to health outcome data and primary care records. Primary care data linkage was achieved through collaboration with clinical commissioning groups, resulting in comprehensive, lifelong primary care prescribing records. G&H participants represent a community with high rates of multimorbidity, leading to elevated levels of medication exposure. These features, combined with complete prescribing histories and genetic data, position the G&H cohort as an ideal resource for the described proof-of-concept study.

### Identification of parent/offspring trios

We used the curated list of parent and offspring trios provided by the G&H team. Trios were identified using the Genes and Health GSA data (44,396 individuals) and cross-linked to the WES 44k data (44,028 individuals). Filtering was performed by the G&H team using PLINK v1.9 to remove Mendelian errors and age discrepancies.^10^

### Analysis of trios

Genetically confirmed parent-offspring trios were mapped to general practitioner-linked medication data to explore medication exposure overlap across generations in 600 trios. The percentage of medications prescribed to offspring that either parent had also been exposed to was calculated, and the offspring’s age was plotted against the percentage of overlap across generations. The most commonly prescribed medications across these trios were identified.

Amitriptyline was prescribed across generations in 96 trios. To investigate its use, a premature discontinuation phenotype for amitriptyline, as a proxy for adverse drug reactions, was curated, defined as discontinuation within three months of the index prescription. UK guidelines recommend that antidepressant efficacy and tolerability be formally reviewed between 4 and 12 weeks after initiation, before committing to long-term treatment or switching agents.^11^

### Statistics and reproducibility

Multivariable logistic regression was employed to determine whether parental discontinuation predicted offspring discontinuation, adjusting for offspring age and gender as covariates. Sensitivity analyses included repeating analyses with a six-month threshold for discontinuation to verify robustness. We assessed the linear relationship between offspring age and the percentage of shared medications with parents using Pearson correlation.

To examine genetic factors, a Fisher's exact test was conducted to assess whether offspring with a two-generation history of early amitriptyline discontinuation were more likely to carry two key loss-of-function variants (rs4244285 (*2) and rs4986893(*3)) in the *CYP2C19* gene, which encodes the Cytochrome P450 2C19 (CYP2C19) enzyme known to metabolise amitriptyline.^12^ These variants, known to affect the metabolism of amitriptyline, have been characterized in this cohort in previous publications, and account for 99% of poor metabolisers in Asian ancestry populations.^13,14^ A diplotype of any combination of these two alleles leads to a genetically predicted poor metaboliser of CYP2C19.

### Ethics

A favourable ethical opinion for the main Genes & Health research study was granted by NRES Committee London - South East (reference 14/LO/1240) on 16 Sept 2014. Informed consent was given by all participants. Queen Mary University of London is the Sponsor.

### Reporting summary

Further information on research design is available in the Nature Portfolio Reporting Summary linked to this article.

## Results

Offspring and parent demographics are shown in Table 1. The average age of the 600 offspring was 32 years old and 61% were female. The average age of the fathers was 64 years old, and the average age of the mothers was 58 years old.

**Table 1:** Age and sex of all 600 trios included in the analysis and the 96 trios in the amitriptyline sub analysis group

*N* participants	600 offspring	1200 parents	96 offspring	192 parents
Age (Mean +/−SD)	32+/−9	Father: 64+/− 10 Mother: 58+/− 9	38+/− 8	Father: 70+/− 9 Mother: 63+/− 8
Male	39%	50%	18%	50%
Female	61%	50%	82%	50%

**Fig. 1:**
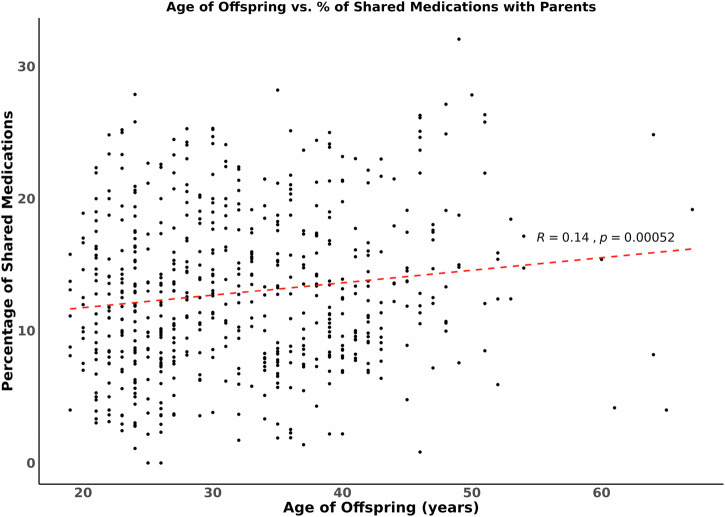
Percentage medication overlap across generations vs age of the offspring. The percentage of prescribed medication to offspring which had also been prescribed to a parent is shown on the *y* axis plotted against the increasing age of offspring on the *x* axis. The correlation is shown with a dotted red line, including the correlation coefficient (*R*).

Medication exposure overlap across two generations in these 600 trios is shown in Table 2. Approximately 13% of the medication prescribed to the offspring, on average, had been prescribed to at least one of the parents (Mean 12.9%, Median 12.5%, SD 6.0%) (Table 2). The percentage overlap across generations is shown plotted against the age of the offspring in Fig. 1. Figure 1 illustrates the general trend that older offspring tend to have slightly increased medication overlap with their parents, though the correlation is modest (*r* = 0.14, *p* = 0.001). The source data for Fig. 1 is in Supplementary Data 1.

**Table 2:** Medication exposure overlap across two generations

	Mean	Median	SD
Number of medications prescribed to offspring	39.2	33.0	26.9
Number of medications prescribed to parents	125.7	116.0	61.0
% Overlap of medications for offspring and parent	12.9	12.5	6.0

*SD* standard deviation.

The top 50 medications most commonly prescribed across generations in these trios are shown in Supplementary Data 2. These are predominantly acute or short-term therapeutics, with antibiotics, vaccines, steroids, and antihistamines being the most frequently used, accounting for 24%, 12%, 10%, and 8% of prescriptions, respectively. Notably, 96 of these trios exhibited cross-generational exposure to the tricyclic antidepressant amitriptyline, which is also indicated to treat some forms of pain and headache.^15^ Premature discontinuation of the medication, as a proxy for adverse drug reactions, was examined across the trios, revealing that 48 offspring (11 males [22.9%], 37 females [77.1%]), or 50% of the offspring cohort prescribed amitriptyline, had discontinued amitriptyline within three months of the index prescription (Supplementary Table 1). Mothers were more frequently prescribed amitriptyline compared to fathers, with 91% of mothers and 57% of fathers (in these 96 trios) receiving the prescription.

Within the trios, 27% of parents had discontinued amitriptyline prematurely within three months, with 15% of mothers and 24% of fathers discontinuing. None of the offspring had both parents prematurely discontinue amitriptyline, leaving 26 trios where at least one parent discontinued the drug prematurely. Among these trios with a parental discontinuation, 16 offspring also prematurely discontinued the medication, amounting to 62% discontinuation in offspring with a parental discontinuation. Logistic regression analysis, adjusting for offspring age and gender, indicated no significant independent association between parental discontinuation and offspring discontinuation of amitriptyline (*p* = 0.275 OR 1.70, CI 0.66–4.51). Sensitivity analysis using a six-month cut-off for premature discontinuation did not alter these findings.

**Fig. 2:**
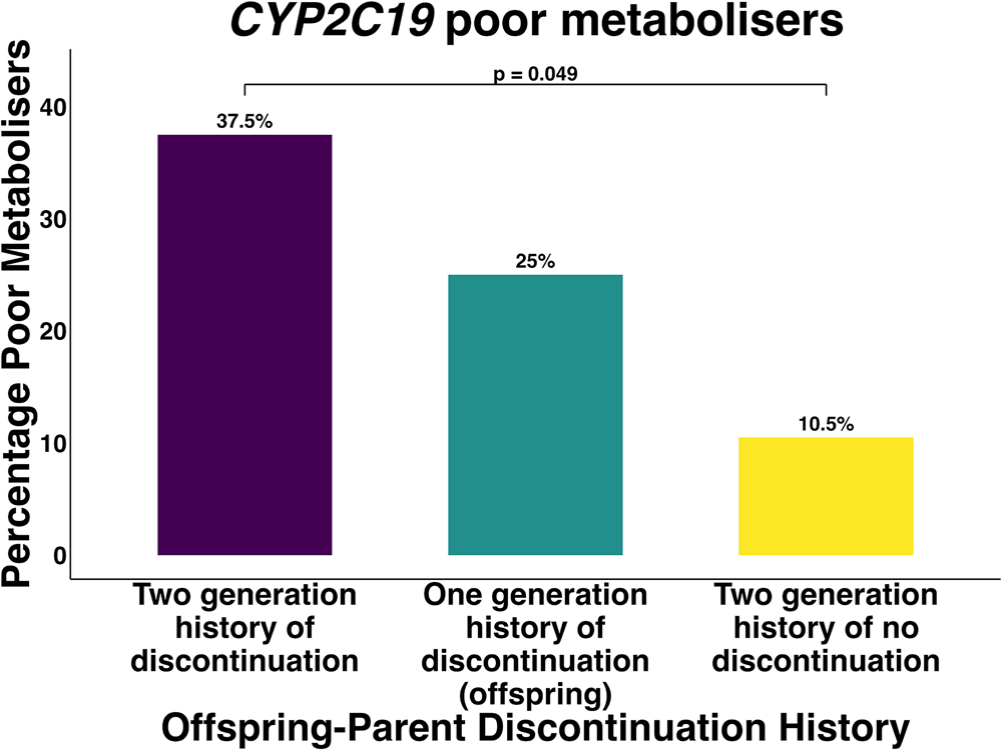
Enrichment of CYP2C19 genetically predicted poor metabolisers in offspring with a two-generation history of early amitriptyline discontinuation. The percentage of CYP2C19 genetically predicted poor metabolisers is shown in offspring with a two-generation history of premature amitriptyline discontinuation, a one generation history of premature discontinuation, and two generations of exposure to the drug with no early discontinuation in either offspring or a parent.

A Fisher's exact test assessed whether offspring with a two-generation history of early amitriptyline discontinuation were more likely to be genetically classified as poor CYP2C19 metabolizers compared to those with a two-generation history of exposure without early discontinuation. Offspring with a two-generation history of early discontinuation showed a significantly higher likelihood of being genetically predicted poor CYP2C19 metabolizers (*p* = 0.049, OR 4.86, CI 1.13–23.45), with 37.5% classified as poor metabolizers compared to 10.5% in the group with a two-generation history of medication exposure but without early discontinuation in either generation (Fig. 2). Sub cohorts with either offspring-only discontinuation or parent-only discontinuation did not significantly differ from the no-discontinuation group in the percentage of poor metabolizers (offspring-only: *p* = 0.126, OR 0.36, CI 0.09–1.32; parent-only: *p* = 0.567, OR Inf, CI 0.17–Inf). The prevalence of variants in these sub-cohorts is shown in Supplementary Table 2.

## Discussion

To our knowledge, this is the first description of cross-generational prescription-medication exposure, in a British South Asian cohort of 600 parent–offspring trios with linked genetic and primary-care prescribing data. Most of these medications are often used acutely, but some are for chronic conditions/medications indicated for long-term use. As ADRs are often badly coded or not coded at all in EHRs, discontinuation phenotypes for chronic use medications can serve for opportunistic large-scale analysis.

This feasibility study, looking at family history of amitriptyline discontinuation, did not show that parental response predicted offspring response. Amitriptyline is a tricyclic antidepressant indicated to treat depression and pain.^15^ It is a tertiary amine and it is metabolised by the hepatic enzyme CYP2C19 to a secondary amine, with less serotonergic activity and more noradrenergic activity.^12^ Loss of function variants in the *CYP2C19* gene which encodes the CYP2C19 enzyme are known to be common in this ancestry population and have been associated with reduced metabolism and consortia recommendations to either avoid or reduce the dose of tertiary amines such as amitriptyline in genetically predicted poor metabolisers (to avoid adverse drug reactions).^12–14^

**Fig. 3:**
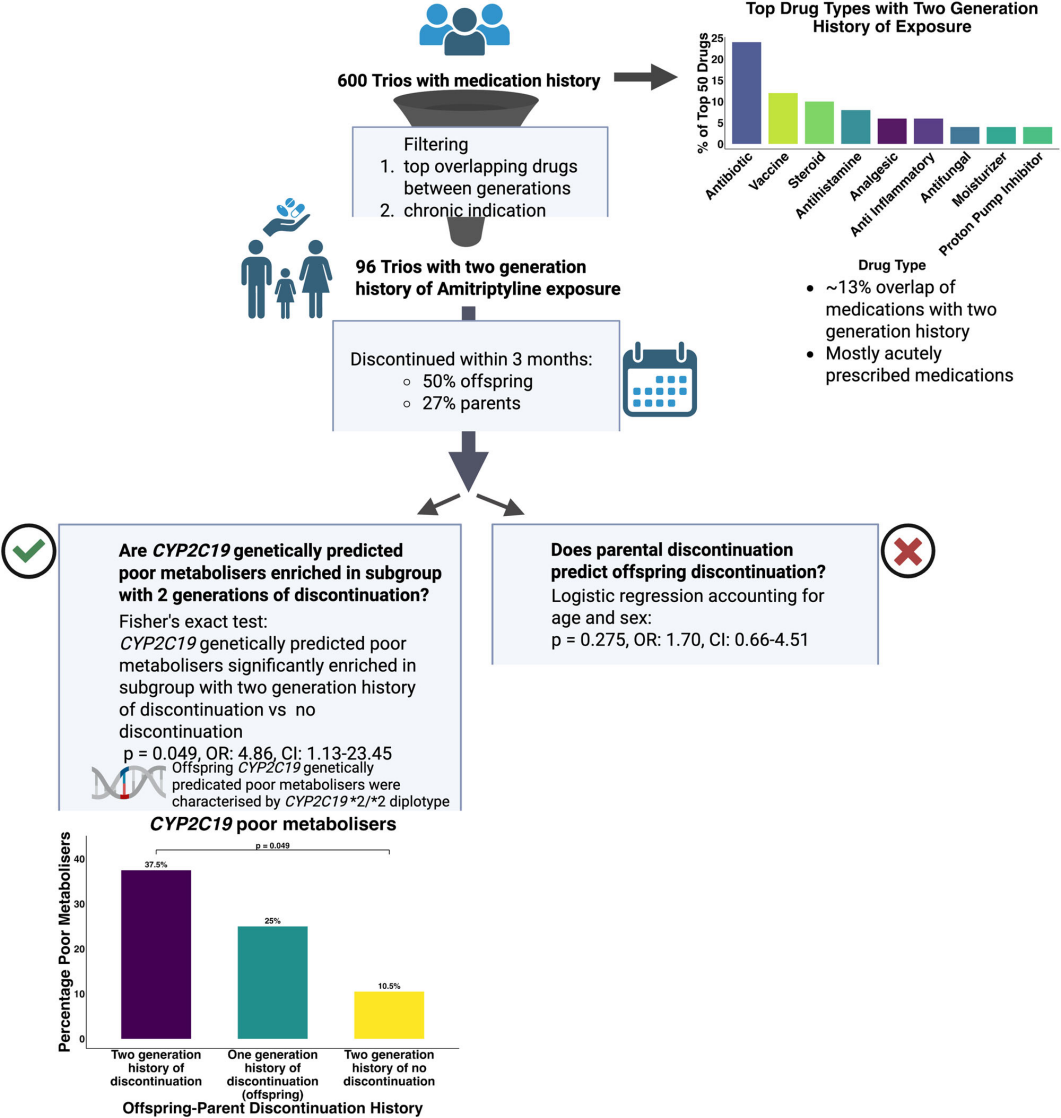
Study overview. 600 family trios of parents and offspring enrolled in the G&H study were identified. Prescription medication patterns across generations were characterised. 96 trios were identified with cross-generational exposure to amitriptyline. An early discontinuation phenotype was curated as an indication of medication intolerance or adverse reaction. Statistical testing was undertaken to assess the relationship between parental and offspring medication discontinuation and genetically predicted metaboliser types. Created in BioRender. marengo, g. (2025) https://BioRender.com/ d7nxfio.

Early discontinuation rates were higher in the offspring than the parental generation and may indicate different generational attitudes, as suggested by prior studies showing higher medication adherence in older patient populations as compared with younger.^16,17^ CYP2C19 genetically predicted poor metabolisers were significantly enriched in the offspring with a two-generational history of early drug discontinuation compared with offspring with a two-generational history of exposure and no early discontinuation. Therefore, a two-generational history of this phenotype could flag individuals who could benefit most from *CYP2C19* PGx testing (Fig. 3). Practically, the use of FHH for guiding PGx testing depends on reliable family medical histories or comprehensive shared medical records, neither of which may be universally available, potentially leading to inequities in clinical practice. If performed on a larger scale across multiple medications with common pharmacokinetic pharmacogenes, such as *CYP2C19*, *CYP2D6*, *CYP2C9*, where data is available, it is reasonable to hypothesize that AI methods may be able to identify progeny with high-risk medication response phenotypes based on family history of medication response or merely discontinuation prescription patterns. While improved characterisation of medication response and ADRs with precise coding would of course improve the resolution, this shows that even a relatively poor resolution discontinuation phenotype with a sample size of 600 trios can show enrichment for variants known to be associated with poor metabolism status in the context of a commonly used tricyclic antidepressant. Prospective integration of family response to medication in clinical history prior to prescribing antidepressions in conjunction with pharmacogenomic testing should be trialled to elucidate possible clinical benefits of this approach.

Though a compelling proof-of-concept study, there are limitations to the real-world data presented. Discontinuation is an imperfect proxy for medication intolerance or inefficacy and we do not have adherence data. We did not include clinical indication or dosage in our analysis, both of which may potentially influence discontinuation rates. However, this could obscure a signal that is present but is unlikely to lead to a false positive finding. Our model did not consider drug-drug interaction data, which can impact on the relevant metabolism pathways and impact on drug metabolism phenotype via phenoconversion. However, if such a crude phenotype opportunistically taken from EHR data with relatively small numbers on a population scale can show real potential utility there is clearly impetus for further investigation in this arena.

Amitriptyline is also metabolised by Cytochrome P450 2D6 (CYP2D6) which is encoded by a highly polymorphic gene, and the use of characterised variants can be used prospectively to guide prescribing.^12^ Due to the structural nature of many of these variants we are not able to call the *CYP2D6* diplotype with confidence from the G&H array or exome data, and we have chosen to focus on *CYP2C19* for this proof-of-concept study. CYP2D6 genetically predicted poor metabolisers are not common in Asian populations and regardless of CYP2D6 metabolism state, CPIC guidance gives an ‘Avoid amitriptyline use’ recommendation for genetically predicted poor CYP2C19 metabolisers.^12^

Our approach omitted gain-of-function alleles (*17) which may also impact on response to amitriptyline. This proof-of-concept study was focused on the predictive value of poor metabolism in the context of the high prevalence of the *2 allele in this population and the stronger evidence base for recommendations based solely on the genetically predicted poor metaboliser type.^12^ Future studies at a larger scale would be better equipped to introduce more variables and should include this variant.

Additionally, as our study specifically analysed individuals of Bangladeshi and Pakistani descent, findings may have limited external validity to other populations. Further studies in diverse and larger cohorts are needed.

## Conclusions

Family history of medication response can highlight individuals who are enriched for pharmacogenomic variants. This is a clinically useful finding, which may flag psychiatric patients with a two-generation history of early amitriptyline discontinuation or intolerance as a priority for pharmacogenomic testing. This is a case study in the potential real-world utility of collecting and analysing family history of medication response.

## Supplementary information

Supplementary Information

Supplementary Data 1

Supplementary Data 2

Reporting Summary

## Data Availability

All Genes & Health data can be accessed by application to the study access team https://www.genesandhealth.org/research/scientists-using-genes-health-scientific-research. The source data for Fig. 1 is available in Supplementary Data 1. All other data are available on request.

## Code availability

The code used for this analysis can be found on github https://github.com/whri-phenogenomics/MedTrioPaper.^18^

## References

[1] Guttmacher, A. E., Collins, F. S. & Carmona, R. H. The family history — more important than ever. *N. Engl. J. Med.* **351**, 2333–2336 (2004). 15564550 10.1056/NEJMsb042979

[2] Six, A. J., Backus, B. E. & Kelder, J. C. Chest pain in the emergency room: value of the HEART score. *Neth. Heart J.* **16**, 191–196 (2008). 18665203 10.1007/BF03086144 PMC2442661

[3] Roden, D. M. & George, A. L. Jr The genetic basis of variability in drug responses. *Nat. Rev. Drug Discov.* **1**, 37–44 (2002). 12119608 10.1038/nrd705

[4] Pirmohamed, M. Pharmacogenomics: current status and future perspectives. *Nat. Rev. Genet* **24**, 350–362 (2023). 36707729 10.1038/s41576-022-00572-8

[5] World Health Organization. Adherence to Long-Term Therapies: Evidence for Action. https://iris.who.int/handle/10665/42682 (World Health Organization, 2003).

[6] McDermott, J. H. et al. Clinical pharmacogenetics implementation consortium guideline for the use of aminoglycosides based on *MT-RNR1* genotype. *Clin. Pharm. Ther.*10.1002/cpt.2309 (2021). 10.1002/cpt.2309 PMC8613315 34032273

[7] Haga, S. B. & Orlando, L. A. Expanding family health history to include family medication history. *J. Pers. Med.* **13**, 410 (2023). 36983592 10.3390/jpm13030410 PMC10053261

[8] Smith, T. R., Kearney, E., Hulick, P. J. & Kisor, D. F. History repeats itself: the family medication history and pharmacogenomics. *Pharmacogenomics* **17**, 669–678 (2016). 27143300 10.2217/pgs-2015-0015

[9] Finer, S. et al. Cohort profile: East London Genes & Health (ELGH), a community-based population genomics and health study in British Bangladeshi and British Pakistani people. *Int. J. Epidemiol.* **49**, 20–21i (2020). 31504546 10.1093/ije/dyz174 PMC7124496

[10] Purcell, S. et al. PLINK: a tool set for whole-genome association and population-based linkage analyses. * Am. J. Hum. Genet.* **81**, 559–575 (2007). 17701901 10.1086/519795 PMC1950838

[11] National Institute for Health and Care Excellence. *Depression in Adults: Treatment and Management* (NICE Guideline NG222, 2022). 35977056

[12] Hicks, J. et al. Clinical pharmacogenetics implementation consortium guideline (CPIC) for *CYP2D6* and *CYP2C19* genotypes and dosing of tricyclic antidepressants: 2016 update. *Clin. Pharm. Ther.* **102**, 37–44 (2017). 10.1002/cpt.597 PMC5478479 27997040

[13] Magavern, E. et al. CYP2C19 genotype prevalence and association with recurrent myocardial infarction in British-South Asians treated with clopidogrel. *JACC Adv.*10.1016/j.jacadv.2023.100573 (2023). 37808344 10.1016/j.jacadv.2023.100573 PMC10550831

[14] Magavern, E. F., van Heel, D. A., Smedley, D. & Caulfield, M. J. *CYP2C19* loss-of-function alleles are not associated with higher prevalence of gastrointestinal bleeds in those who have been prescribed antidepressants: Analysis in a British-South Asian cohort. *Br. J. Clin. Pharm.* **89**, 3432–3438 (2023). 10.1111/bcp.15762 37143396

[15] National Institute for Health and Care Excellence. BNF: Amitriptyline hydrochloride. https://bnf.nice.org.uk/drugs/amitriptyline-hydrochloride/#indications-and-dose (British National Formulary, 2025).

[16] Ge, L., Heng, B. H. & Yap, C. W. Understanding reasons and determinants of medication non-adherence in community-dwelling adults: a cross-sectional study comparing young and older age groups. *BMC Health Serv. Res.* **23**, 905 (2023). 37620970 10.1186/s12913-023-09904-8 PMC10464472

[17] Reading, S. R. et al. Risk factors for medication non-adherence among atrial fibrillation patients. *BMC Cardiovasc. Disord.* **19**, 38 (2019). 30744554 10.1186/s12872-019-1019-1 PMC6371431

[18] Marengo, G. MedTrioPaper [source code]. Zenodo. 10.5281/zenodo.16539280 (2025).

