# Supplementary material for: Family health history and pharmacogenomics show cross generation premature amitriptyline discontinuation is associated with CYP2C19 loss of-function enrichment
Source: Commun Med (Lond). 2025 Nov 13;5:437. doi: 10.1038/s43856-025-01156-3 (PMC12615714; doi:10.1038/s43856-025-01156-3)
Supplement: Supplementary file 1 — Supplementary Information [file 43856_2025_1156_MOESM1_ESM.pdf]

Supplementary Table 1. Amitriptyline exposure and discontinuation stratification by gender.

Generation	N	Prescribed N (%)	Discontinued ≤ 3 months N (%)
Offspring	96	Total 96 (100 %)<br>Male 17 (18%)<br>Female 79 (82 %)	Total 48 (50 %)<br>Male 11 (23%)<br>Female 37 (77 %)
Mothers	96	87 (91 %)	13 (15 %)
Fathers	96	55 (57 %)	13 (24 %)

Supplementary Table 2. Offspring subgroup *CYP2C19* diplotype and predicted metaboliser phenotype. This study did not characterise the *CYP2C19**17 allele because clinical guidance for patients carrying that allele is dependent on CYP2D6 status as well, which we did not assess[12]. Therefore, the table below will potentially misclassify rapid or ultrarapid metabolizers as normal metabolizers.

Subgroup	N Offspring	Diplotype	Phenotype	N	%
Two-generation no discontinuation	38	*1/*1	Normal metabolizer	17	44.7
		*1/*2	Intermediate metabolizer	17	44.7
		*2/*2	Poor metabolizer	4	10.5
Two-generation discontinuation	16	*1/*1	Normal metabolizer	6	37.5
		*1/*2	Intermediate metabolizer	4	25.0
		*2/*2	Poor metabolizer	6	37.5
Only offspring discontinued	32	*1/*1	Normal metabolizer	14	43.8
		*1/*2	Intermediate metabolizer	10	31.3
		*2/*2	Poor metabolizer	8	25.0

Only parent discontinued	10	*1/*1	Normal metabolizer	6	60.0
		*1/*2	Intermediate metabolizer	4	40.0
		*2/*2	Poor metabolizer	0	0.0

Genes & Health Research Team

Eamonn Maher, Shabana Chaudhary, Joseph Gafton, Karen A Hunt, Shapna Hussain, Kamrul Islam, Mohammed Bodrul Mazid, Elizabeth Owor, Jessry Russell, Nishat Safa, John Solly, Marie Spreckley, David A Van Heel, Jan Whalley, Ishevanhu Zengeya, Emily Mantle, Shaheen Akhtar, Samina Ashraf, Dan Mason, John Wright, Daniel MacArthur, Michael Simpson, Richard C Trembath, Gerome Breen, Raymond Chung, Sang Hyuck Lee, Omar Asgar, Joanne Harvey, Karen Tricker, Caroline Winckley, Hanifa Khatun, Amna Asif, Claudia Langenberg, Grainne Colligan, Ceri Durham, Bill Newman, Ahsan Khan, Hilary Martin, Teng Heng, Matt Hurles, Vivek Iyer, Georgios Kalantzis, Vladimir Ovchinnikov, Iaroslav Popov, Klaudia Walter, Panos Deloukas, David Collier, Ana Angel, Saeed Bidi, Fabiola Eto, Sarah Finer, Chris Griffiths, Sam Hodgson, Benjamin M Jacobs, Rohini Mathur, Caroline Morton, Asma Qureshi, Stuart Rison, Annum Salman, Miriam Samuel, Moneeza K Siddiqui, Daniel Stow, Sabina Yasmin, Julia Zöllner, Sheik Dowlut
